# Urinary neopterin of wild chimpanzees indicates that cell-mediated immune activity varies by age, sex, and female reproductive status

**DOI:** 10.1038/s41598-021-88401-6

**Published:** 2021-04-29

**Authors:** Jacob D. Negrey, Verena Behringer, Kevin E. Langergraber, Tobias Deschner

**Affiliations:** 1 Department of Anthropology, Boston University, Boston, MA 02215 USA; 2 Department of Pathobiological Sciences, University of Wisconsin-Madison, 2015 Linden Dr., Madison, WI 53706 USA; 3 Endocrinology Laboratory, German Primate Center, Leibniz Institute for Primate Research, 37077 Göttingen, Germany; 4 School of Human Evolution and Social Change, Arizona State University, Tempe, AZ 85287 USA; 5 Institute of Human Origins, Arizona State University, Tempe, AZ 85287 USA; 6 Interim Group Primatology, Max Planck Institute for Evolutionary Anthropology, Deutscher Platz 6, 04103 Leipzig, Germany

**Keywords:** Ecology, Evolution, Immunology, Biomarkers

## Abstract

The study of free-living animal populations is necessary to understand life history trade-offs associated with immune investment. To investigate the role of life history strategies in shaping proinflammatory cell-mediated immune function, we analyzed age, sex, and reproductive status as predictors of urinary neopterin in 70 sexually mature chimpanzees (*Pan troglodytes*) at Ngogo, Kibale National Park, Uganda. In the absence of clinical signs of acute infectious disease, neopterin levels significantly increased with age in both male and female chimpanzees, as observed in humans and several other vertebrate species. Furthermore, males exhibited higher neopterin levels than females across adulthood. Finally, females with full sexual swellings, pregnant females, and post-reproductive females, the oldest individuals in our sample, exhibited higher neopterin levels than lactating females and cycling females without full swellings. Variation in females’ neopterin levels by reproductive status is consistent with post-ovulatory and pregnancy-related immune patterns documented in humans. Together, our results provide evidence of ample variation in chimpanzee immune activity corresponding to biodemographic and physiological variation. Future studies comparing immune activity across ecological conditions and social systems are essential for understanding the life histories of primates and other mammals.

## Introduction

Activation of the immune system, a key component of somatic maintenance and survival, likely poses trade-offs with development and reproduction^1^ and may even accelerate organismal senescence^2,3^. Consequently, investment in immune function is hypothesized to vary according to its relative importance in maximizing reproductive success in a particular life stage or ecological context^4^. Unravelling these complex interactions requires that we study organisms within the ecological conditions to which they are adapted. Ecoimmunology^5^, in which the immune system is understood in the light of evolutionary and ecological pressures, prioritizes the study of free-ranging, genetically diverse, and energetically limited populations exposed to an array of naturally occurring pathogens. An ecoimmunological perspective has been productively applied to various vertebrate species, including humans^6^. For instance, substantial differences have been noted between industrialized and foraging and hunter-gatherer populations, illuminating both local ecological influences and shared physiological mechanisms. Notably, the Tsimané, a forager-horticulturalist population in Bolivia, exhibit higher inflammatory biomarkers throughout the life course than do individuals living in the USA^7,8^, a pattern that likely reflects the pathogen-dense environment inhabited by the Tsimané^9,10^. Yet, despite ecological disparities, rates of physiological dysregulation between the Tsimané and industrialized populations are similar, suggesting conserved rates of organismal senescence across human populations^11^. As these results demonstrate, distinguishing between universal and context-dependent physiological patterns, and understanding the complex trade-offs between immune function and reproduction, requires special attention to environment.

Despite the proliferation of ecoimmunological research on various vertebrate taxa, ecoimmunological studies of nonhuman primates, and apes in particular, remain relatively rare. This scarcity is largely due to ethical and practical complications surrounding data collection in these species. While analyses of captive populations provide invaluable insights into primate health (e.g., evidence for conserved patterns of age-related physiological dysregulation across species^12^), it is unclear to what the extent these results apply to wild populations—given, for instance, notable behavioral^13^, anatomical^14^, and developmental^15^ differences between captive and wild chimpanzees (*Pan troglodytes*). To fully understand how nonhuman primate immune systems develop, function, and senesce in the environments to which they are adapted, we must consider data from wild primate populations.

### Biodemography and immune function

Several key biodemographic variables, including age, may elucidate immune function’s role in the life histories of primates. As long-lived animals enter advanced age and experience loss of fecundity, they experience diminished immune efficacy. This process, termed immunosenescence, entails “inflammaging”—i.e., age-related chronic low-grade inflammation that weakens adaptive immune function^16^—and increased susceptibility to infectious diseases^17^. Inflammaging is a particularly well-documented component of aging in humans and captive nonhuman primates^18,19^. Yet, it has been hypothesized that inflammaging and other immunosenescent processes result from novel lifestyle attributes arising from evolutionarily recent demographic shifts and therefore do not represent universal aging processes^20^. Although studies of immunosenescence in wild nonhuman mammals remain relatively rare^21^, age-related increases in inflammatory biomarkers have been observed in several free-living mammal populations, including those of roe deer (*Capreolus capreolus*)^22^, Soay sheep (*Ovis aries*)^23^, and mandrills (*Mandrillus sphinx*)^24^, as well as a nonindustrialized human population^7^. These results suggest that inflammaging is more widespread than previously thought.

A second important biodemographic variable is sex. Given that reproductive rates generally vary more in males than in females, health and longevity are more strongly linked to reproductive success in females than males^25^. Consequently, for females of many species, investment in immune function should maximize longevity^26^. In most mammals, life expectancy is shorter in males than females^27^, which is due, in part, to females’ generally more robust responsiveness to infection^26^. For most immune measures, females show greater quantities and activity of both innate and adaptive immune components, although males often exhibit higher levels of proinflammatory cytokines^26^. These sex differences have important consequences for health. For instance, across vertebrate species, males appear to exhibit higher parasite burdens than females^28^. This pattern applies to humans: males appear more susceptible to infectious disease than females and exhibit higher rates of infection-related mortality^29^. Therefore, considering the various genetic, hormonal, and environmental differences underlying these patterns as well as the consequences for health and longevity, sex is a critical variable for understanding immune function^26^, especially in the framework of life history.

A third variable of interest is female reproductive state. To support both sperm survival and embryonic implantation in the endometrium, T helper cell type 1 (Th1) immune activity is largely downregulated in the female reproductive tract around and following ovulation^30^, although the local activity of some cytokines may be necessary for uterine remodeling and successful implantation^31^. Consequently, in humans, susceptibility to bacterial and viral infection increases during the ovulatory and post-ovulatory period. For instance, frequency of bacterial vaginosis increases in the second week of the menstrual cycle, prior to ovulation^32^, while vulnerability to human immunodeficiency virus infection is elevated for seven to ten days following ovulation^33^. Furthermore, much like fertilization and embryonic implantation, pregnancy itself poses considerable immunological challenges, given that immune activity must be carefully modulated to prevent fetal loss^34^. While patterns may vary by cell or cytokine type, pregnant females generally exhibit downregulated Th1 immune responses^34^ and are more susceptible to various infections^35^.

### Chimpanzee life history and immune function in the wild

Chimpanzees are valuable subjects for ecoimmunological inquiry for several reasons. For instance, in contrast to many model organisms, chimpanzees are long-lived^36^. Given evidence that pace-of-life can shape immune investment^37,38^, immunological data from wild chimpanzees can contribute to wider discussions of life history trade-offs when reproductive lifespans encompass decades. Furthermore, the close genetic proximity between humans and chimpanzees can elucidate both shared and distinct immune mechanisms^39^, with consequences for human evolutionary biology. Unfortunately, immune function is difficult to analyze in wild great apes, from whom only noninvasive measurements may be routinely collected for ethical and logistical reasons. These complications are compounded by the unknown pathogenicity of many infectious agents hosted by wild chimpanzees. For instance, while eukaryotic gastrointestinal parasites of wild chimpanzees have received considerable attention^40,41^, the costs associated with many such parasites remain unclear^42^.

In place of infectious agents, the noninvasive measurement of immune biomarkers in excreta may capture an organism’s immune activity. A promising and recently validated noninvasive measure of cell-mediated immune activation in primates, including chimpanzees, is neopterin^43,44^. Often used in humans as a biomarker of inflammation^45^, neopterin is produced from the oxidation of 7,8-dihydroneopterin, which is released by monocytes and macrophages following stimulation by interferon gamma^46^. Given its association with inflammation, neopterin concentrations in blood and urine are positively correlated with disease severity and the likelihood of mortality in humans^47^ and chimpanzees^48^. In the absence of clinical signs of disease, chimpanzees exhibited 43% higher urinary neopterin concentration in the wild than in captivity^49^. This pattern indicates that wild populations face greater pathogenic burdens when apparently subclinical, corresponding to higher pathogen exposure and an absence of routine medical care in the wild^49^.

To investigate immune function’s role in wild chimpanzee life histories, we examined age, sex, and female reproductive status as predictors of urinary neopterin excretion in generally healthy chimpanzees from the long-lived^36^ Ngogo community of Kibale National Park, Uganda. If inflammaging results primarily from recent lifestyle shifts (e.g., decreased physical activity), the increased proinflammatory markers observed in aging members of industrialized human populations and captive nonhuman primates should not be present in wild primate populations. In support of this hypothesis, a recent study of the Kanyawara chimpanzee community, also located in Kibale, found that urinary neopterin levels did not increase across the life course^50^. However, among three past-prime individuals who died during the study period, neopterin increased as the time to death decreased^50^. Furthermore, data on infectious agent abundances suggest that wild chimpanzees experience notable immunosenescence, as older individuals exhibit infections at higher frequencies and burdens. In both the Ngogo and Kanyawara communities, apparently healthy males, although not females, exhibited increased gastrointestinal viral richness with age^51^, and among females, gastrointestinal helminth richness increased with age^52^. Furthermore, at Kanyawara, older chimpanzees of both sexes were more likely to exhibit clinical signs of respiratory disease, although young adult males were more likely to exhibit signs than were age-matched females^53^. It therefore seems likely that wild chimpanzees experience a pronounced immunosenescence which should be reflected by increases in urinary neopterin throughout adulthood.

Existing data with which to assess immune variation corresponding to sex and reproduction in wild chimpanzees are limited. While female chimpanzees live longer than males in both captivity^54^ and in the wild^36^, evidence for sex biases in chimpanzee immune function is mixed. Among sanctuary chimpanzees, older males exhibited greater risk of inflammatory diseases with advanced age than did females^55^. Yet, in wild chimpanzees at Kanyawara, there were no sex differences in urinary neopterin concentrations^50^, nor did reproductive status predict observations of respiratory illness among females^53^. There is, however, evidence of pregnancy-related immunomodulation in wild chimpanzees, as late-term pregnant females at Ngogo and Kanyawara shed more gastrointestinal parasites than other females^52^ and in Taï National Park, Côte d’Ivoire, were more likely to test positive for malaria^56^. Therefore, existing evidence concerning the roles of sex and reproductive status in shaping chimpanzee immune activity is largely inconclusive.

## Methods

### Ethics declaration

This non-invasive research was approved by the Uganda National Council for Science and Technology and Uganda Wildlife Authority, and formally exempt from review by Boston University’s Institutional Animal Care and Use Committee. We adhered to all national and international guidelines for the study of wild chimpanzees.

### Study subjects and sample collection

We collected observational data and biological samples from the Ngogo chimpanzee community from February 2016 to July 2017. At the beginning of the study period, there were approximately 204 individuals in the community, all of whom were individually identifiable. With the exception of individuals born after 1995, when continuous long-term study of this community began, ages were estimated from both pedigrees and morphological features^36^. We sampled 70 sexually mature chimpanzees (females: N = 36; males: N = 34). Females ranged in age from 14 to 67 years and males from 16 to 54 years.

Urine samples were opportunistically collected during daily focal follows of chimpanzees. Urine was pipetted from leaves^57^ or plastic sheets^58^ and transferred to collection vials. Samples contaminated with fecal matter were discarded. Samples were stored on ice in a thermos until the collector returned to camp, at which point samples were stored in a solar-powered freezer at − 20 °C. Samples were transported on dry ice from Uganda to Germany and then stored at − 80 °C at the Max Planck Institute for Evolutionary Anthropology in Leipzig.

### Hormone analyses

Urine samples were analyzed with a commercially available neopterin enzyme immunoassay kit (Neopterin ELISA, Ref. RE59321, IBL International GmbH, Hamburg, Germany), which exhibits 2% cross-reactivity with 7,8-dihydroneopterin. This minor cross-reactivity is unlikely to affect results, especially given that neopterin and 7,8-dihydroneopterin are closely correlated and have similar clinical utilities^59^. The assay was previously validated for use with chimpanzee urine^43^. Samples were diluted from 1:10 to 1:2000 using the provided assay buffer to bring concentrations into the assay’s working range. The assay was then performed following the manufacturer’s instructions, with samples, standards, and controls measured in duplicate. Samples were remeasured if duplicate optical density values differed by more than 10% or if the measurements were outside the assay’s working range. Intra-assay coefficients of variance (CVs) for high and low quality controls (included in the kit) were 9.0% and 9.7%, respectively; inter-assay CVs for high and low quality controls were 8.4% and 11.3% (N = 20 plates), respectively.

To correct for variation in urine concentration, the specific gravity (SG) of each sample was measured with a digital refractometer (TEC, Ober-Ramstadt, Germany). While creatinine has often been used to correct for urine concentration, SG is preferable to creatinine when analyzing samples from individuals of highly variable body size and age^60,61^. The concentration of urine in each sample was adjusted for SG following Miller, et al. ^62^. To avoid overestimating the effects of sample dilution, we excluded all samples with an SG below 1.003 (N = 8 samples) and above 1.050 (N = 8 samples). Furthermore, to avoid the confounding effects of severe acute immune challenge on neopterin levels^43,48,50^, we excluded samples collected during an outbreak of respiratory disease^63^ and from individuals otherwise exhibiting clinical signs of acute disease and injury (N = 72 samples), leaving 502 samples for statistical analyses. Final sample sizes for each age and sex class, as well as mean sample sizes for individual chimpanzees, are presented in Supplementary Table S1.

### Statistical analyses

All statistical analyses were completed in R version 4.0.3^64^. We ran two main models, both of which had urinary neopterin as the response variable. Model 1 addressed neopterin’s relationships with age and sex, and contained samples from both males and females. Model 2 addressed neopterin’s relationship with female reproductive status and only included samples from females. We included several additional variables in each model that likely influence neopterin levels. First, we included the time of day at which the sample was collected. While some prior studies of genus *Pan* do not report a diurnal effect on neopterin excretion^43,48^, such an effect has been observed in humans^65^ and in Kanyawara chimpanzees^50^, with a peak in the early morning and low levels in the afternoon. In addition, to control for seasonal variation in neopterin levels^66^, we included the date of sample collection. Finally, we included chimpanzee identity as a random effect to control for multiple sampling of individuals.

Because urinary neopterin might exhibit nonlinear relationships with age, time of day, and season, we first implemented Model 1 as a general additive model (GAM) using the “gam” function in package mgcv^67^. We included the individual’s sex as a parametric predictor and the following smooth terms as cubic regression splines: age, age by sex, time of day, and date. We also included chimpanzee identity as a random effect to control for multiple sampling of individuals. However, we found that the age by sex variable was not significant, and the age and time of day variables produced estimated degrees of freedom equal to 1, indicating that these predictors exhibited a linear relationship with age. The results of this model are presented in Supplementary Table S2.

Given the linearity of the age and time of day predictors, we instead implemented linear mixed models (LMMs) with Gaussian error structures and fitted with restricted maximum likelihood using the “lmer” function in package lmerTest^68^ as modified from lme4^69^. We included age, sex, and time of day as fixed effects. We addressed the nonlinearity of larger temporal trends by also including the sine and cosine of the Julian date (multiplied by 2pi and divided by 365.25) as fixed effects^66,70^. Finally, we included random slopes where possible, although problems with model convergence largely prohibited the inclusion of random slopes. In Model 1, we included the cosine of the Julian date as a random slope. We did not include any random slopes in Model 2.

In Model 2, we analyzed variation in female chimpanzees’ urinary neopterin levels by reproductive status. We included reproductive status as a categorical variable with six levels: cycling without a sexual swelling, cycling with a partial sexual swelling, cycling with a full sexual swelling, pregnant, lactating, and post-reproductive. The model was run six times to assess each reproductive state as the reference category. Pregnancy was determined by one of two methods. First, we classified a female as pregnant if the sample collection date fell between the estimated dates that she conceived and gave birth. To generate a conservative estimate of the conception date, we counted back 253 days from the estimated date of birth, corresponding to the mean plus two standard deviations of pregnancy duration in captive chimpanzees^71^. Alternatively, we used a commercial pregnancy test. If a female exhibited a sexual swelling within 4 weeks (28 days) of her estimated conception date, she was classified as cycling. As per a modification of Caro, et al. ^72^, females were classified as putatively post-reproductive if they had previously given birth but had not done so within 7.9 years—i.e., the mean plus two standard deviations of successful, closed interbirth intervals in the Ngogo community.

We Box-Cox transformed neopterin concentrations for analysis^73^ and *z*-transformed all continuous fixed effects (except Julian date) to a mean of 0 and a standard deviation of 1. We used Satterthwaite approximations in package lmerTest^68^ to estimate degrees of freedom and produce p values with reliably low type I error rates^74^. For each model, we calculated marginal and conditional effect sizes—i.e., the variation explained by the fixed effects and the variation explained by both random and fixed effects, respectively—using the “r.squaredGLMM” function in MuMIn^75^. To satisfy the assumptions of normality and homoscedasticity, we assessed normality of model residuals with Shapiro–Wilk tests^76^ and inspection of residual plots, histograms, and QQ plots^77^. We examined collinearity by checking variance inflation factors (VIFs) using the “vif” function in package car^78^. Although all VIFs in Model 1 were < 2, we found relatively high VIFs (> 3) in Model 2 indicating collinearity between reproductive status and age. Because this model was primarily intended to assess reproductive status rather than age, we removed the age variable from this model. Notably, the model with both reproductive status and sex was largely indistinguishable from the final model, with two important exceptions: when both terms were included in the model, neopterin did not exhibit a significant relationship with either the post-reproductive category (β = 0.098, SE = 0.073, p = 0.197) or age (β = 0.048, SE = 0.030, p = 0.125). All VIFs in the final model were < 2.

## Results

**Figure 1:**
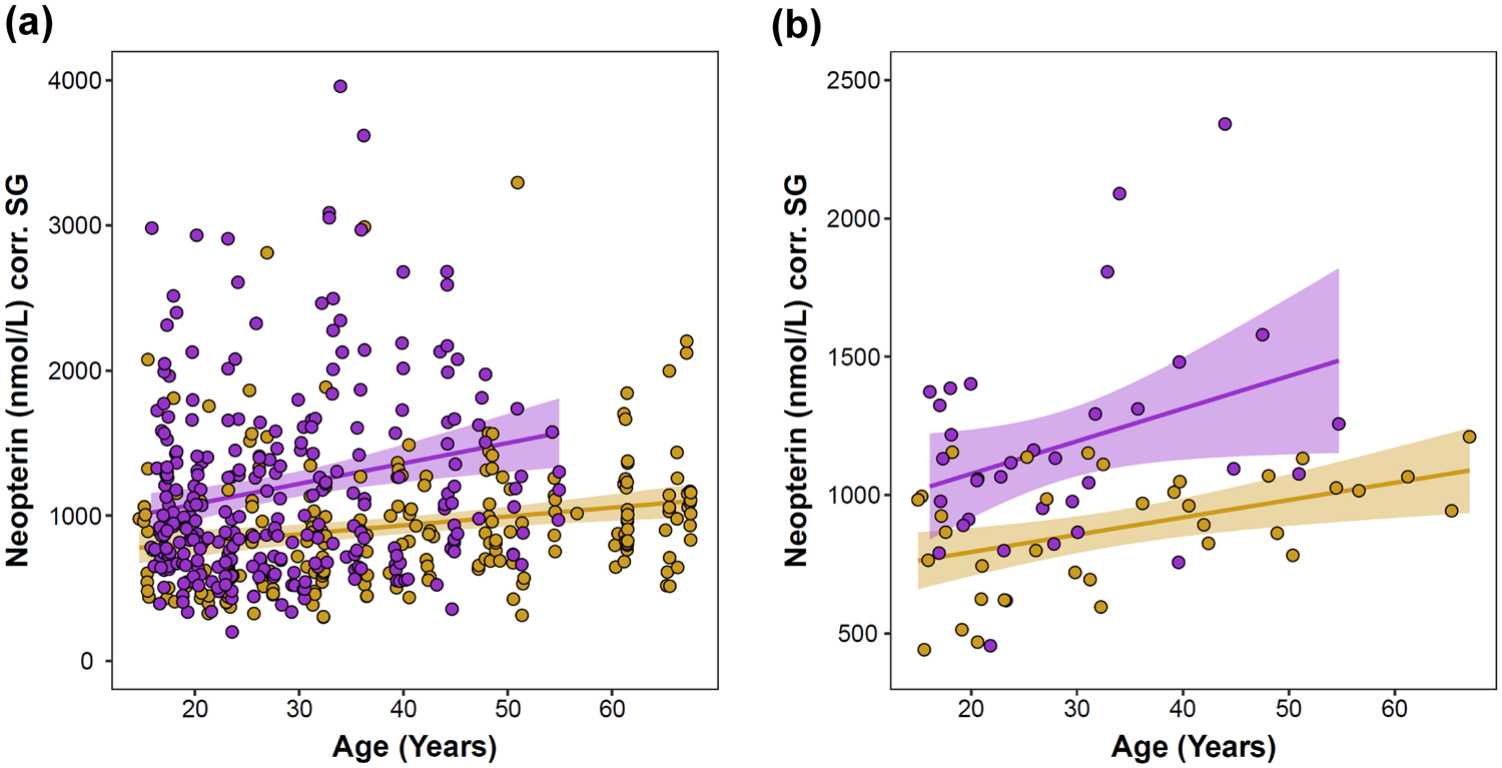
Urinary neopterin concentrations of wild chimpanzees by subject age and sex. Both the raw data points **(a)** and subject means **(b)** are presented. Shading around the regression lines indicates 95% confidence intervals. Yellow = females, purple = males. Two data points in Fig. 2a are greater than the maximum limit of the y-axis and are therefore not visible.

In apparently healthy adult chimpanzees, urinary neopterin varied from 199.8 to 6407.3 nmol/L corr. SG (mean ± SD = 1081.0 ± 638.8 nmol/L corr. SG). As indicated by general additive modeling, neopterin exhibited a significant linear relationship with age (edf = 1.000, p = 0.013; Supplementary Table S2). The effect of age did not significantly differ between males and females (edf = 1.000, p = 0.893; Supplementary Table S2). A subsequent linear mixed model, which yielded marginal and conditional R^2^ values of 0.238 and 0.369, respectively, reinforced these results: neopterin levels modestly but significantly increased with age in both males and females (Table 1; Fig. 1). To assess the role of sample sizes in the detection of this age-related pattern, we ran a post-hoc test in which we retained samples from a random sample of 36 individuals—roughly half the total number of individuals sampled—and re-ran the LMM with the reduced sample set. We repeated this procedure 10,000 times and found that the age variable remained significant in 63.67% of the generated models. Following prior research^50^, we conducted two additional post-hoc tests to further test the importance of sample size and determine if age-related effects were consistent across the adult lifespan. We separately analyzed urine samples collected from “prime” (< 35 years) and “past-prime” (≥ 35 years) individuals. In both models, the effect of age was substantially reduced: Urinary neopterin did not significantly vary by age in either the prime (β = 0.007, SE = 0.012, p = 0.535; Supplementary Table S3) or past-prime sample sets (β = 0.007, SE = 0.004, p = 0.082; Supplementary Table S4).

**Table 1:** Predictors of urinary neopterin levels in 502 samples from 70 sexually mature chimpanzees (N_males_ = 34, N_females_ = 36).

Term	β	SE	DF	p
Intercept	− 1.373	0.011		
**Age (years)**	**0.026**	**0.008**	**58.646**	**0.002**
**Sex (male)**	**0.053**	**0.015**	**71.596**	**0.001**
**Time of day**	− **0.024**	**0.005**	**488.821**	** < 0.001**
Sine (Julian date)	0.001	0.007	484.998	0.847
**Cosine (Julian date)**	**0.065**	**0.010**	**67.875**	** < 0.001**

*SE*  standard error, *DF* degrees of freedom estimated by Satterthwaite approximation.

Bold font denotes significance. Please note that urinary neopterin levels were Box–Cox transformed prior to analysis.

Neopterin levels significantly varied by sex: Males exhibited higher urinary neopterin levels than females across adulthood (Table 1; Fig. 1). Concentrations in males ranged from 199.8 to 6407.3 nmol/L corr. SG (mean ± SD = 1198.8 ± 737.1 nmol/L corr. SG) and in females from 301.2 to 3294.8 nmol/L corr. SG (mean ± SD = 933.6 ± 448.7 nmol/L corr. SG).

**Figure 2:**
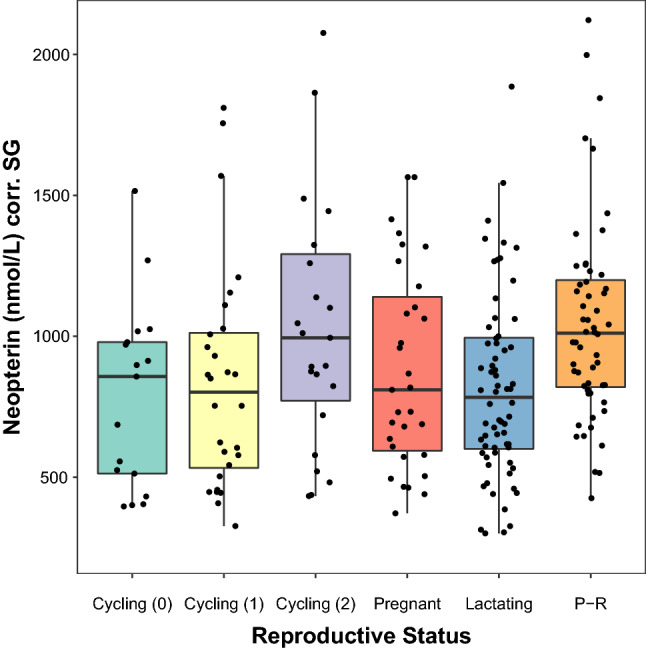
Urinary neopterin levels of female chimpanzees as predicted by reproductive status. Reproductive statuses include cycling without a sexual swelling (“Cycling 0”), cycling with a partial sexual swelling (“Cycling 1”), cycling with a full sexual swelling (“Cycling 2”), pregnant, lactating, and post-reproductive (“P-R”). Upper and lower bounds of the boxes demarcate the 75th and 25th percentiles, respectively, and bold horizontal lines represent medians.

Neopterin levels also significantly varied by reproductive status in female chimpanzees (marginal R^2^ = 0.272; conditional R^2^ = 0.462). Paired comparisons of neopterin levels by reproductive status are presented in Table 2; full model results are available in Supplementary Table S5. We excluded subject age from this analysis due to collinearity with reproductive status. (See Methods for additional information.) Lactating females and cycling females without swellings exhibited the lowest neopterin levels, while pregnant females exhibited modestly higher neopterin levels (Table 2; Fig. 2). Cycling females with full sexual swellings and putatively post-reproductive females exhibited the highest urinary neopterin levels, differing significantly from lactating females and cycling females who were not fully swollen (Table 2; Fig. 2).

**Table 2:** Paired comparisons (β ± standard error) of urinary neopterin levels by reproductive status in 36 female chimpanzees.

Reference Level	Cycling (0)	Cycling (1)	Cycling (2)	Pregnant	Lactating	Post-reproductive
Cycling (0)		0.118 ± 0.070^†^	0.276 ± 0.074***	0.227 ± 0.079**	0.074 ± 0.061	0.252 ± 0.070**
Cycling (1)	−0.118 ± 0.070^†^		0.159 ± 0.062*	0.109 ± 0.071	−0.044 ± 0.054	0.134 ± 0.062*
Cycling (2)	−0.276 ± 0.074***	−0.159 ± 0.062*		−0.050 ± 0.076	−0.203 ± 0.060***	−0.024 ± 0.068
Pregnant	−0.227 ± 0.079**	−0.109 ± 0.071	0.050 ± 0.076		−0.153 ± 0.065*	0.026 ± 0.073
Lactating	−0.074 ± 0.061	0.044 ± 0.054	0.203 ± 0.060***	0.153 ± 0.065*		0.178 ± 0.053**
Post-reproductive	−0.252 ± 0.070**	−0.134 ± 0.062*	0.024 ± 0.068	−0.026 ± 0.073	−0.178 ± 0.053**	

Values were derived from a linear mixed model in which reproductive status was a categorical predictor; the model was run six times so that each reproductive status could serve as the reference level. Cycling females are distinguished by sexual swelling size: 0 = no swelling, 1 = partial swelling, and 2 = full swelling. Please note that urinary neopterin levels were Box–Cox transformed prior to analysis.

p-values: *** < 0.001, ** < 0.01, * < 0.05, † < 0.10.

## Discussion

We analyzed variation in urinary concentrations of the cell-mediated biomarker neopterin by age, sex, and reproductive status in wild chimpanzees and found several notable patterns. First, urinary neopterin concentrations increased with age in both males and females. Similar age-related increases in neopterin concentrations have been observed in humans^79^ and captive Barbary macaques (*Macaca sylvanus*)^80^, indicating immune dysregulation. Our results add to a growing body of knowledge in wild mammals^22–24^ challenging the hypothesis that such “inflammaging” is due to recent human demographic and lifestyle shifts. Instead, our results suggest that inflammaging could be common across mammalian taxa, especially in long-lived animals, and persists perhaps because selective forces for late-life traits are generally weak^81^. If increased inflammation early in life bolsters reproductive success, and fertility declines late in life, there is minimal selective pressure to mitigate the late-life health consequences of inflammation. In the present study, post-reproductive females, the oldest females in the sample, exhibited generally higher neopterin levels than most other sexually mature females. As these individuals will no longer reproduce and do not seemingly contribute to the reproductive success of their offspring through food provisioning, coalitionary support, or other forms of helping behavior^82^, there is likely little selective pressure to mitigate chronic inflammation in old age. Furthermore, in humans, inflammatory dysregulation underlies several non-communicable diseases most often experienced in middle and late life. These notably include rheumatoid arthritis, the manifestation of which corresponds to elevated neopterin levels^83^. The age-related increase in neopterin observed in our data suggest that wild chimpanzees may experience similar health conditions. Additional data from wild nonhuman primates, and mammals more broadly, will illuminate the prevalence of such immunosenescent processes and inflammatory diseases.

Importantly, we cannot fully disentangle the effects of immune dysregulation from those of pathogenic infection *resulting from dysregulation*. An investigation of viral fecal shedding in this community suggests that females, unlike males, do not exhibit increased viral richness with age^51^. However, a separate study of female chimpanzees in this population found an age-related increase in gastrointestinal helminth and protozoan richness as well as the prevalence and intensity of *Oesophagostomum*^52^. Given that the responsiveness of neopterin to gastrointestinal parasites is not fully understood^80,84^, data from non-viral intracellular infections of known pathogenicity (e.g., malaria) will help distinguish the effects of systemic dysregulation from those of infectious disease itself. Likewise, it is unclear whether old male chimpanzees exhibit high neopterin levels independently of infectious disease burden. Therefore, future studies will need to integrate measurements of pathogens and immune biomarkers to assess their independent relationships with age, using multiple biomarkers from different branches of the immune system. Such direct comparisons of pathogenic infection and immune biomarkers will help clarify the role of infectious disease in driving the higher neopterin levels observed in old chimpanzees.

Second, as recently observed in wild mandrills^24^, we found that males exhibited higher neopterin levels than females, supporting the hypothesis that male and female strategies result in different degrees of cellular and proinflammatory immune responses. While the costs of gestation and lactation are immense^85^, current evidence does not suggest that greater reproductive success imposes heavy immune burdens on female chimpanzees. Rather, in Kibale chimpanzees, more fertile females exhibited lower levels of gastrointestinal parasites^52^. In contrast, the reproductive strategies of male chimpanzees may require extensive somatic repair for several reasons. Male chimpanzees frequently engage in intrasexual contest competition in which they sustain physical injuries^86^ that stimulate inflammatory immune responses^50^. Furthermore, male chimpanzees are more gregarious than females^87^, which likely increases exposure to infectious agents. Indeed, more gregarious primates harbor greater loads of both useful^88^ and pathogenic^89^ agents. Yet another explanation is that physiological disparities drive sex differences in disease susceptibility. As previously indicated, in most measures, females exhibit stronger innate *and* adaptive immune responses^26^. These differences result, in part, from differences in circulating hormone concentrations, including the immunomodulatory steroid hormone testosterone^90^.

Whatever the cause(s), sex differences in chimpanzee neopterin levels may indicate a physiological disparity that contributes to the longer life expectancies of females^36^. Studies of humans suggest that lower inflammatory responsiveness and/or increased anti-inflammatory activity across adulthood are linked to late life survival and health^91^. By contributing to the pathogenesis of various diseases, inflammation decreases functional and absolute longevity in systems throughout the body^92^. Therefore, in the absence of severe pathogenic infection, low levels of inflammation likely facilitate “healthy” aging and late-life survival^7^. In humans, such effects are perhaps reflected in cross-population analyses: Foraging populations with high burdens of infectious disease and inflammation across the lifespan exhibit lower life expectancies than industrialized populations^93^. Consequently, we propose that the high levels of urinary neopterin exhibited by male chimpanzees at Ngogo may reflect a trade-off: The physiological processes underlying successful male reproductive strategies, separate from those of females, may result in relatively shorter lives. Male strategies may result in high levels of inflammation that augment reproductive success while reducing lifespan. However, it is important to note that current evidence for sex differences in aging rates in many mammalian species is inconclusive^27^. Long-term data on inflammatory profiles in wild chimpanzees will help determine if such immunological processes contribute to longevity in this species.

Critically, studies adjusting for urine diluteness with specific gravity do not report sex differences in the urinary neopterin of captive chimpanzees^43^ or Barbary macaques^80^. In contrast, a human study using creatinine to adjust urinary neopterin levels found that females exhibited higher levels than males^94^. However, disparities in muscle mass between males and females yield sex differences in creatinine excretion rates and can generate misleading results when creatinine is used as a urinary correction factor^61^. Given the absence of sex differences in captive populations, the sex difference observed in Ngogo chimpanzees is likely driven by behavioral and ecological variables experienced by free-living animals, and highlights the importance of studying immune function “in the wild”. In other words, the impact of sex-dependent reproductive strategies on chimpanzee neopterin levels are perhaps only observable in wild populations where these strategies are fully implemented.

Third, we found variation in female neopterin levels pertaining to reproductive status. In particular, females with full sexual swellings (a morphological feature that signals probability of ovulation and stimulates mating^95^) and pregnant females exhibited higher neopterin levels than lactating females. The increase in neopterin levels corresponding to full sexual swellings may have resulted for several reasons. Infectious disease exposure is likely elevated in fully tumescent females due to high rates of sexual contact as well as temporary expansion of social contact networks^96^. Furthermore, as previously indicated, physiological variation throughout the menstrual cycle (e.g., increases in estradiol around and following ovulation) modulates immune function and disease susceptibility^97^. Physical aggression may also contribute to this pattern: cycling females are more likely to receive aggression from males, especially when fully swollen^98^. Prior research indicates that urinary neopterin in wild chimpanzees is responsive to severe injury^50^. It is worth noting that neopterin may be less sensitive to physical injury than to intracellular infection; while data from humans suggest that neopterin increases in response to cardiovascular^99^, liver^100^, and knee surgery^101^, macaques appear to exhibit very weak neopterin responsiveness to surgical interventions^102^. None of these results are necessarily inconsistent with our own, however, as the neopterin levels we observed in fully swollen females, while relatively high, are much lower than those reported for chimpanzees enduring severe acute infectious disease^48^.

We also observed a mild pregnancy-related increase in neopterin levels, a pattern which ostensibly contradicts the expectation of pregnancy-induced Th1 immunosuppression. Our finding may reflect the various and competing physiological demands present during pregnancy. As previously indicated, neopterin levels in humans often increase in the third trimester of healthy pregnancies, presumably due to fetal antigenic stimulation^103^, while elevated neopterin levels may be observed in any trimester of a complicated pregnancy^104–106^. Furthermore, Th1 suppression by pregnant females may increase susceptibility to intracellular infection, which in turn may stimulate a cell-mediated immune response and result in higher neopterin levels^104^. A comparable push-and-pull between pregnancy and infection is likely experienced by chimpanzees, given prior evidence that pregnant females are more susceptible to infection than females in other reproductive states^52,56^. At present, we cannot adequately distinguish between factors contributing to higher neopterin in pregnant chimpanzees, which would require extensive physiological and parasitological data. Even so, our results suggest that pregnancy-related immunomodulation is a rich subject for future study in wild nonhuman primates.

Importantly, our results contrast in several ways with those from a recent study of the spatially proximate Kanyawara chimpanzee community^50^. While urinary neopterin predicted the approach to death in Kanyawara chimpanzees, it did not increase throughout adulthood as it did at Ngogo^50^. Furthermore, neopterin concentrations did not vary by sex at Kanyawara^50^. While many of the technical specifications for the two studies were comparable, including use of the same commercial immunoassay and similar sample sizes (i.e., 409 and 502 urine samples at Kanyawara and Ngogo, respectively), there are notable differences in study design. In contrast to our 18 month collection period, the Kanyawara study analyzed samples collected from individual chimpanzees over approximately five years^50^. Perhaps the mixed-longitudinal approach of the Kanyawara study effectively circumvented the biases present in cross-sectional and short-term studies. On the other hand, our study incorporated more chimpanzees, especially those of advanced age: the Kanyawara data included nine individuals over 35 years and none ≥ 60 years, whereas the Ngogo data contained 23 individuals over 35 years and three individuals ≥ 60 years. The larger number of individuals in our sample may have enabled detection of age-related effects, as randomly halving the number of individuals in our sample reduced the likelihood of observing an age-related effect to 64%.

However, there are also important ecological differences between Ngogo and Kanyawara that may explain differences in neopterin excretion. For instance, ripe fruit availability is greater at Ngogo^107^. Chimpanzees at Ngogo consequently exhibit higher energy balances than those at Kanyawara^108^. As diet affects immune function^109^, energetic differences between Ngogo and Kanyawara may drive inter-community differences in immune function and disease susceptibility throughout the life course. Indeed, females at Kanyawara shed more gastrointestinal parasites than females at Ngogo^52^. More importantly, life expectancies for chimpanzees in these two communities are markedly different: 57% of Ngogo chimpanzees live to age 30 compared to 18% of Kanyawara chimpanzees^36^. As suggested by González, et al.^50^, the absence of age-related increases in urinary neopterin excretion at Kanyawara may reflect a survivorship bias: the relatively few individuals who survive to old age may be those with particularly low levels of inflammation and oxidative stress. In contrast, perhaps many types of individuals at Ngogo survive to old age, not just those with unusually low levels of inflammation and oxidative stress.

In conclusion, we found variation in wild chimpanzee urinary neopterin excretion corresponding to important biodemographic variables including age, sex, and female reproductive status. Thus, even in the absence of discernible infectious disease, urinary neopterin exhibited considerable biodemographic variation in a long-lived primate species. Our results provide ecologically valid information on chimpanzee immune activation pertinent to life history and further demonstrate the utility of urinary neopterin in the noninvasive study of wild chimpanzees.

## Supplementary Information

Supplementary Tables.

## Data Availability

Original data and R code have been deposited on Figshare (https://doi.org/10.6084/m9.figshare.13118639).
