# Supplementary material for: Urinary neopterin of wild chimpanzees indicates that cell-mediated immune activity varies by age, sex, and female reproductive status
Source: Sci Rep. 2021 Apr 29;11:9298. doi: 10.1038/s41598-021-88401-6 (PMC8085242; doi:10.1038/s41598-021-88401-6)
Supplement: Supplementary file 1 — Supplementary Tables. [file 41598_2021_88401_MOESM1_ESM.pdf]

Urinary neopterin of wild chimpanzees indicates that cell-mediated immune activity varies by age, sex, and female reproductive status

Jacob D. Negrey[1,2*], Verena Behringer[3], Kevin E. Langergraber[4,5], and Tobias Deschner[6**]

[1]Department of Anthropology, Boston University, Boston, MA, 02215, USA
[2]Department of Pathobiological Sciences, University of Wisconsin-Madison, Madison, WI, 53706, USA
[3]Endocrinology Laboratory, German Primate Center, Leibniz Institute for Primate Research, Göttingen, 37077, DE
[4]School of Human Evolution and Social Change, Arizona State University, Tempe, AZ, 85287, USA
[5]Institute of Human Origins, Arizona State University, Tempe, AZ, 85287, USA
[6]Interim Group Primatology, Max Planck Institute for Evolutionary Anthropology, Leipzig, 04103, DE

***Corresponding author:** negrey@wisc.edu
****Corresponding author:** deschner@eva.mpg.de

Supplementary Table S1. Summary of urine samples by chimpanzee age and sex. "Prime" and "past-prime" refer to chimpanzees aged <35 years and ≥35 years, respectively. Values in parentheses indicate the mean and standard deviation of urine samples per individual chimpanzee.

**Sex**	**Prime** (N = 21 females, 26 males)	**Past-Prime** (N = 15 females, 8 males)	**Overall** (N = 36 females, 34 males)
Females	100 (4.76 ± 3.60)	123 (8.20 ± 6.35)	223 (6.19 ± 5.15)
Males	205 (7.89 ± 3.94)	74 (9.25 ± 4.71)	279 (8.21 ± 4.10)
Total	305 (6.49 ± 4.07)	197 (8.57 ± 5.74)	502 (7.17 ± 4.75)

Supplementary Table S2. Results of a generalized additive model predicting urinary neopterin levels in male and female chimpanzees. edf = estimated degrees of freedom. Bold font indicates statistical significance.

Parametric Coefficients	β	SE	p
Intercept	-1.386	0.011	**<0.001**
Sex	0.059	0.022	**0.009**
Smooth Terms	edf	p	
Age x Sex	1.000	0.893	
Age	1.000	**0.013**	
Time of Day	1.000	**<0.001**	
Date	7.411	**<0.001**	
Subject ID (Random Effect)	29.725	**<0.001**	

Supplementary Table S3. Results of a post-hoc linear mixed model examining urinary neopterin in "prime" individuals aged <35 years (N = 47 individuals, 305 urine samples). Bold font indicates statistical significance.

Predictor	β	SE	DF	p
Intercept	-2.240	0.022		
Age (Years)	0.007	0.012	39.804	0.535
**Sex (Male)**	**0.075**	**0.026**	**47.218**	**0.006**
**Time of Day**	**-0.032**	**0.009**	**292.081**	**<0.001**
Sine (Julian Date)	0.004	0.013	290.132	0.774
**Cosine (Julian Date)**	**0.095**	**0.017**	**45.686**	**<0.001**

Supplementary Table S4. Results of a post-hoc linear mixed model examining urinary neopterin in "past-prime" individuals aged ≥35 years (N = 23 individuals, 197 urine samples). Bold font indicates statistical significance.

Predictor	β	SE	DF	p
Intercept	-0.206	0.004		
Age (Years)	0.007	0.004	16.660	0.082
Sex (Male)	0.013	0.007	21.650	0.070
**Time of Day**	**-0.006**	**0.002**	**190.600**	**0.006**
Sine (Julian Date)	-0.001	0.003	189.400	0.781
**Cosine (Julian Date)**	**0.015**	**0.004**	**19.970**	**0.002**

Supplementary Table S5. Results for six variants of a linear mixed model predicting neopterin by female reproductive status. "Reproductive status" is a categorical variable with six levels, and each level acted as the reference level in one of the model variants. Bold font indicates statistical significance.

Reference Level	Predictor	β	SE	DF	p
Lactating	Intercept	-7.241	0.031		
	Reproductive Status - Cycling (0)	-0.074	0.061	76.848	0.231
	Reproductive Status - Cycling (1)	0.044	0.054	82.979	0.421
	**Reproductive Status - Cycling (2)**	**0.203**	**0.060**	**137.883**	**0.001**
	**Reproductive Status - Pregnant**	**0.153**	**0.065**	**97.271**	**0.022**
	**Reproductive Status - Post-Reproductive**	**0.178**	**0.053**	**13.697**	**0.005**
	**Time of Day**	**-0.082**	**0.014**	**210.264**	**<0.001**
	Sine (Julian Date)	-0.042	0.025	196.857	0.094
	**Cosine (Julian Date)**	**0.150**	**0.035**	**41.875**	**<0.001**
Cycling (0)	Intercept	-7.315	0.053		
	Reproductive Status - Cycling (1)	0.118	0.070	209.819	0.094
	**Reproductive Status - Cycling (2)**	**0.276**	**0.074**	**193.350**	**<0.001**
	**Reproductive Status - Pregnant**	**0.227**	**0.079**	**124.583**	**0.005**
	**Reproductive Status - Post-Reproductive**	**0.252**	**0.070**	**30.657**	**0.001**
	Reproductive Status - Lactating	0.074	0.061	76.848	0.231
	**Time of Day**	**-0.082**	**0.014**	**210.264**	**<0.001**
	Sine (Julian Date)	-0.042	0.025	196.857	0.094
	**Cosine (Julian Date)**	**0.150**	**0.035**	**41.875**	**<0.001**
Cycling (1)	Intercept	-7.197	0.047		

	**Reproductive Status - Cycling (2)**	**0.159**	**0.062**	**209.359**	**0.012**
	Reproductive Status - Pregnant	0.109	0.071	120.438	0.129
	**Reproductive Status - Post-Reproductive**	**0.134**	**0.062**	**24.283**	**0.040**
	Reproductive Status - Lactating	-0.044	0.054	82.979	0.421
	Reproductive Status - Cycling (0)	-0.118	0.070	209.819	0.094
	**Time of Day**	**-0.082**	**0.014**	**210.264**	**<0.001**
	Sine (Julian Date)	-0.042	0.025	196.857	0.094
	**Cosine (Julian Date)**	**0.150**	**0.035**	**41.875**	**<0.001**
Cycling (2)	Intercept	-7.039	0.054		
	Reproductive Status - Pregnant	-0.050	0.076	116.122	0.514
	Reproductive Status - Post-Reproductive	-0.024	0.068	37.035	0.722
	**Reproductive Status - Lactating**	**-0.203**	**0.060**	**137.883**	**0.001**
	**Reproductive Status - Cycling (0)**	**-0.276**	**0.074**	**193.350**	**<0.001**
	**Reproductive Status - Cycling (1)**	**-0.159**	**0.062**	**209.359**	**0.012**
	**Time of Day**	**-0.082**	**0.014**	**210.264**	**<0.001**
	Sine (Julian Date)	-0.042	0.025	196.857	0.094
	**Cosine (Julian Date)**	**0.150**	**0.035**	**41.875**	**<0.001**
Pregnant	Intercept	-7.088	0.061		
	Reproductive Status - Post-Reproductive	0.026	0.073	33.073	0.729
	**Reproductive Status - Lactating**	**-0.153**	**0.065**	**97.271**	**0.022**
	**Reproductive Status - Cycling (0)**	**-0.227**	**0.079**	**124.583**	**0.005**
	Reproductive Status - Cycling (1)	-0.109	0.071	120.438	0.129
	Reproductive Status - Cycling (2)	0.050	0.076	116.122	0.514
	**Time of Day**	**-0.082**	**0.014**	**210.264**	**<0.001**
	Sine (Julian Date)	-0.042	0.025	196.857	0.094
	**Cosine (Julian Date)**	**0.150**	**0.035**	**41.875**	**<0.001**
Post-Reproductive	Intercept	-7.063	0.046		
	**Reproductive Status - Lactating**	**-0.178**	**0.053**	**13.697**	**0.005**
	**Reproductive Status - Cycling (0)**	**-0.252**	**0.070**	**30.657**	**0.001**
	**Reproductive Status - Cycling (1)**	**-0.134**	**0.062**	**24.283**	**0.040**
	Reproductive Status - Cycling (2)	0.024	0.068	37.035	0.722
	Reproductive Status - Pregnant	-0.026	0.073	33.073	0.729
	**Time of Day**	**-0.082**	**0.014**	**210.264**	**<0.001**
	Sine (Julian Date)	-0.042	0.025	196.857	0.094
	**Cosine (Julian Date)**	**0.150**	**0.035**	**41.875**	**<0.001**
